# iAKA-CIoT: An Improved Authentication and Key Agreement Scheme for Cloud Enabled Internet of Things Using Physical Unclonable Function

**DOI:** 10.3390/s22166264

**Published:** 2022-08-20

**Authors:** Kisung Park, Youngho Park

**Affiliations:** 1 Blockchain & Big Data Research Department, Electronics and Telecommunications Research Institute, Daejeon 34129, Korea; 2 School of Electronics Engineering, Kyungpook National University, Daegu 41566, Korea

**Keywords:** key establishment, Internet of Things (IoT), physical unclonable function, authentication

## Abstract

The Internet of Things (IoT) with cloud services are important functionalities in the latest IoT systems for providing various convenient services. These cloud-enabled IoT environments collect, analyze, and monitor surrounding data, resulting in the most effective handling of large amounts of heterogeneous data. In these environments, secure authentication with a key agreement mechanism is essential to ensure user and data privacy when transmitting data between the cloud server and IoT nodes. In this study, we prove that the previous scheme contains various security threats, and hence cannot guarantee essential security requirements. To overcome these security threats, we propose an improved authentication and key agreement scheme for cloud-enabled IoT using PUF. Furthermore, we evaluate its security by performing informal, formal (mathematical), and simulation analyses using the AVISPA tool and ROR model. The performance and security properties of our scheme are subsequently compared with those of other related schemes. The comparison confirms that our scheme is suitable for a practical cloud-enabled IoT environment because it provides a superior security level and is more efficient than contemporary schemes.

## 1. Introduction

The Internet of Things (IoT) and advanced communication technologies are opening up a novel networking paradigm that connects various devices to a public network. By 2025, the number of IoT devices and their market size are estimated to increase to approximately 30 billion [1] and 1.6 trillion [2], respectively. With the expansion of IoT infrastructure, IoT-based smart systems can support social networks in various areas, such as telemedicine, finance, smart grids, intelligent transport systems, and businesses. In these environments, IoT devices analyze the surrounding circumstances, collect data, and send them to service providers to provide various IoT services to users. However, IoT devices generally have limited computing power and storage resources, and do not handle a large amount of heterogeneous data.

Cloud-enabled IoT is known to be the most effective system for handling massive amounts of data generated by IoT devices [3]. In cloud-enabled IoT, a cloud server (CS) has sufficient ability to handle massive amounts of data and has the required storage capability for providing services. IoT devices transfer the collected data by monitoring the surrounding circumstances to utilize the storage and computing power of the CS. Thus, the CS collects IoT data and analyzes it to provide cost-effective and convenient services. Cloud-enabled IoT with communication technologies has become extremely important in human life, and thus the need for security and privacy has become essential for users. This is because of the various sensitive information that IoT data contains, such as health, finance, location, and behavior. Moreover, the Internet is an open channel that causes severe security issues. An adversary can easily forge or intercept data transmitted in an open channel and access user data stored in the CS. Therefore, it is necessary to authenticate entities that attempt to access data.

Numerous authenticated key agreement (AKA) schemes have been presented to guarantee user privacy and data security [4,5,6,7,8,9,10,11]. However, these schemes do not resist physical capture attacks using differential power analysis because IoT devices are not equipped with tamper-proof modules. Although some schemes assume that the devices in their scheme are equipped with tamper-proof modules, they do not present detailed tamper-resistant techniques to prevent physical capture attacks.

A physically unclonable function (PUF) [12] is a novel solution for preventing physical capture attacks targeting devices. The PUF module extracts the unique value corresponding to the inputs from an integrated circuit (IC) that is deployed during the manufacturing process. It has strong and valuable properties, such as tamper-proofing, unpredictable results, and low power consumption, which can be applied to lightweight authentication and identification protocols. In recent years, several PUF-based AKA schemes have been proposed [13,14,15] to ensure the security of the physical layer. In PUF-based AKA schemes, the PUF module can generate the secret value using challenge-response methods from IC which has different physical characteristics. After performing a fuzzy extractor for correcting the noise of a PUF value, it can be applied for AKA schemes as a secret parameter.

In this paper, we cryptanalyze the security flaws of previous schemes and propose an improved AKA scheme for cloud-enabled IoT using a challenge-response-based PUF, called iAKA-CIoT. Additionally, we analyze its security using formal (mathematical) and informal analyses, and conduct a comparative analysis on iAKA-CIoT and other contemporary schemes. Finally, we simulate our scheme to prove that it is secure against potential attacks.

### Motivations and Contributions

The main goal of this study was to propose an improved AKA scheme for IoT using PUF to overcome the security threat of the previous scheme [6,10,11,16,17]. In the previous schemes, an attacker can easily disguise a legal user and compute a correct session key between the participants of the protocol. Moreover, the devices of their scheme can be easily compromised by an attacker using physical capture attack. In our AKA scheme, an adversary cannot compromise the IoT devices because they are protected by PUF modules. We perform informal and formal (mathematical) security analysis, which proves that our scheme meets the essential security requirements and session key security in a threat model. We also conducted a formal simulation analysis using the “automated validation of internet security protocols and applications” (AVISPA) [18] to prove its security and suitability for deployment in an open channel. Finally, the comparative analysis is carried out to evaluate performances and security properties compared with the related schemes.

The organization of this paper is as follows. Section 2, Section 3 and Section 4 discuss the related works, preliminaries and a review of the scheme proposed by Bhuarya et al., respectively. Section 5 presents the security weaknesses of the aforementioned scheme. In Section 6, we propose an improved AKA scheme for IoT using PUF to overcome the security weaknesses of previous schemes. Subsequently, we present the formal-, informal-security and simulation analyses in Section 7. Section 8 presents a comparative analysis of the related schemes. Finally, the conclusion is furnished in Section 9.

## 2. Related Works

In the last decade, several studies have been conducted to guarantee user and data privacy in IoT [4,5,6,7,8,9,10,11,13,14]. In 2014, Islam and Biwas [4] proposed a multi-factor authentication method using elliptic curve cryptosystems (ECCs) to provide secure communication for cloud computing. However, Sarvabhatla and Vorugunti [5] showed that the scheme proposed by Islam and Biwas did not prevent offline password guessing, replay, and user impersonation, and subsequently presented an enhanced ECC-based authentication scheme. However, their scheme is inefficient owing to its high computational cost. In 2015, Kalra and Sood [5] proposed an AKA scheme for cloud-enabled IoT using an ECC. However, in 2017, Kumari et al. [6] showed the security flaws of the Kalra and Sood Schemes and presented an AKA scheme using ECC to resolve these issues. Chaudhry et al. [7] and Chang et al. [8] simultaneously proposed an ECC-based remote user AKA scheme to provide secure mutual AKA. However, in 2019, Mo et al. [9] identified that the scheme proposed by Chaudhry et al. [7] did not resist smart-card loss attacks. Karuppiah et al. [10] proposed a remote AKA for cloud environments. However, Bhuarya et al. [11] pointed out that the aforementioned scheme did not prevent a password-guessing attack and did not achieve user anonymity and secure mutual authentication (SMA). Bhuarya et al. [11] cryptanalyzed the scheme proposed by Kumari et al. [6] and proposed an improved ECC-based AKA for cloud-based IoT. In 2022, Qureshi and Munir [13] also proposed a PUF-based robust authentication and key agreement scheme, and Wang et al. [14] proposed PUF-based authentication scheme with blockchain for wireless sensor network to prevent physical capture attacks. Although many schemes have been proposed, they do not prevent physical capture attacks or have a high communication cost while others simply do not consider them at all, which causes critical security issues.

## 3. Preliminaries

### 3.1. Threat Model

We adopted the Dolev–Yao (DY) threat model [19] to evaluate the security of the cryptographic protocols, including the assumptions proposed by Bhuarya et al. According to the adopted model, an adversary can control all messages transmitted in a public network. Additionally, an adversary can easily guess the identity or password but cannot guess them simultaneously in polynomial time. Moreover, an adversary cannot speculate on the secret parameters (secret key, nonce, random number, etc.) in polynomial time because of its large size. Finally, an adversary can obtain data stored in embedded devices that are not equipped with detailed tamper-proof techniques [20,21,22].

### 3.2. Physical Unclonable Function

A PUF [12] is a physically unclonable one-way function constructed from a semiconductor as an integrated circuit. PUF is based on challenge-response methods and allows for the identification and authentication of the user. In PUF, *c* is a challenge and is the input, and its unique response *r* is illustrated as r=PUF(c). Although the same input is provided, PUF returns an inconsistent output. The PUF has following properties:

(1) Unclonable: There is no function $PUF^{\prime }\left(c\right)$ satisfying PUF(c)=PUF(c), and the probability of duplicating the same result in polynomial time is negligible.
(2) Computable and unpredictable: PUF(c)=r is easily computed; however, it is infeasible to correctly guess *r* of the PUF() corresponding to *c* in polynomial time.

### 3.3. Fuzzy Extractor

The PUF response r=PUF(c) is not perfect because of its susceptibility to surrounding conditions and noise. Therefore, it cannot be utilized in cryptographic protocols as a secret parameter. To correct the noise or errors, we utilize a fuzzy extractor [23,24] that can recover a uniform PUF response *r*. A fuzzy extractor consists of the two following functions.

(1) Generation function Gen: Gen(c)=(a,h), where *c*, *a*, and *h* are the input value, return value, and auxiliary string, respectively.
(2) Reproduction function Rep: Rep=(c,h), where *c* and *h* are the noisy input value and auxiliary string, respectively. Rep can recover the correct *a* from *c* and helper string *h*.

## 4. Review of Bhuarya et al. Scheme

This section reviews the scheme proposed by Bhuarya et al. [11] to demonstrate their security limitations. The scheme consists of three phases: initial, registration, and login and authentication. The notations used in this paper are presented in Table 1.

### 4.1. System Setup Phase

This phase is executed by the CS to set up the initial parameters for the system. The CS selects a large prime number *p*, elliptic curve equation $y^{2}=x^{3}+ax+b$ over the finite field $Z_{p}$, and elements $a,b\in Z_{p}$, where a,b satisfy the condition $4a^{3}+27b^{2}\neq 0$. *G* and *O* are the base points of the elliptic curve and the point at infinity, respectively, where n·G=O. The CS then generates a secret key $MX_{CS}$ and broadcasts the initial public parameters.

### 4.2. Registration Phase

In this phase, embedded devices $ED_{i}$ register themselves with the CS through a secure network to use the CS services. The detailed steps of this phase are as follows.

(1) $ED_{i}$ chooses the identity $ID_{i}$ and password $PW_{i}$. It then computes $I_{i}=h(ID_{i}||PW_{i})$ and sends it to the CS via a secure channel.
(2) After receiving $\left\{I_{i}\right\}$, the CS selects a random number $rn_{s}$ and computes a pseudo identity $PID_{i}=h(rn_{s}||ID_{CS}\left|\right|I_{i})⊕ID_{CS}$ for $ED_{i}$. Afterwards, the CS computes the cookie $C_{k}=h(rn_{s}||MX_{CS}\left|\right|E_{t}||PID_{i})$, $C_{k}^{\prime }=C_{k}\cdot G$, $R_{i}=rn_{s}⊕h(MX_{cs}||PID_{i})$, $A_{i}=h(rn_{s}⊕h(MX_{CS}||PID_{i})⊕I_{i}⊕C_{k}^{\prime })$, and $A_{i}^{\prime }=A_{i}\cdot G$. The CS computes $t_{i}=R_{i}⊕MX_{CS}$, $a_{i}=A_{i}⊕MX_{CS}$, and expiration time $e_{t}=E_{t}$, and then stores it with $PID_{i}$ and sends $\left\{PID_{i},C_{k},R_{i}\right\}$ to $ED_{i}$ through a secure channel. If $C_{k}$ is expired, $E_{t}$ is updated to $E_{t}^{\prime }$ and computes a new cookie $C_{k}=h(rn_{s}||MX_{CS}\left|\right|E_{t}^{\prime }||PID_{i})$.
(3) Finally, $ED_{i}$ stores $PID_{i}$, $R_{i}$, $C_{k}$ with $I_{i}$ in a memory.

### 4.3. Login and Authentication Phase

In this phase, the CS and $ED_{i}$ authenticate each other, which is executed via a public channel. The detailed steps of this phase are as follows.

(1) A user inputs their identity $ID_{i}$ and password $PW_{i}$, and then $ED_{i}$ computes $I_{i}^{*}=h(ID_{i}||PW_{i})$ and checks if $I_{i}^{*}\overset{?}{=}I_{i}$. If it is valid, $ED_{i}$ chooses a random number $rn_{1}$, a current timestamp $T_{1}$, and computes $P_{1}=rn_{1}\cdot G$, $P_{2}=h\left(rn_{1}\cdot C_{k}^{\prime }\right)$, $E=PID_{i}⊕R_{i},K=h\left(P_{1}\right)⊕PID_{i}$, and $Y=h(P_{1}||P_{2}||K||T_{1})$. Then, $ED_{i}$ sends the login request $\left\{E,P_{1},Y,T_{1}\right\}$ to CS.
(2) Upon receiving the login request from $ED_{i}$, the CS checks the timestamp validity, computes $PID_{i}=E⊕R_{i}$, and finds $PID_{i}$ in the database.
(3) If it exists, the CS calculates $rn_{s}=R_{i}⊕h(MX_{CS}||PID_{i})$, $C_{k}=h(rn_{s}||MX_{CS}\left|\right|E_{t}||PID_{i})$, $K=h(P_{1})$, $P_{2}=h\left(P_{1}\cdot C_{k}\right)$, and $Y^{*}=h(P_{1}\left|\right|P_{2}\left||K|\right|T_{1})$, and then verifies that $Y^{*}$ is equal to *Y*. If it is correct, the CS chooses a random number $rn_{2}$ and a current timestamp $T_{3}$ and calculates $P_{3}=rn_{2}\cdot G$, $P_{4}=rn_{2}\cdot A_{i}^{\prime }$, and $S=h(P_{3}||P_{4}||T_{3})$. Subsequently, the CS sends the response messages $\left\{S,P_{3},T_{3}\right\}$ to $ED_{i}$.
(4) After receiving $\left\{S,P_{3},T_{3}\right\}$ from CS, $ED_{i}$ calculates $A_{i}=h\left(R_{i}⊕I_{i}⊕C_{k}^{\prime }\right)$, $P_{3}^{*}=P_{3}\cdot A_{i}$ and $S^{*}=h(P_{3}^{*}\left|\right|P_{4}\left|\right|T_{3})$, and then verifies that $S^{*}\overset{?}{=}S$ and the timestamp is valid. If this is correct, $ED_{i}$ generates the session key $SK=h(rn_{1}\cdot P_{3}||PID_{i}||T_{4}||A_{i})$ and $V_{i}=h(\left(rn_{1}\cdot C_{k}^{\prime }\right)\left||SK\right)$, and then sends the messages $\left\{V_{i},T_{4}\right\}$ to CS.
(5) The CS checks the validity of the timestamp and generates the session key $SK^{*}=h(rn_{2}\cdot P_{1}||PID_{i}\left|\right|T_{4}\left|\right|A_{i})$ and $V_{i}^{*}=h((P_{1}\cdot C_{k}||SK^{*})$. Then, the CS verifies that $V_{i}^{*}$ is equal to $V_{a}$. If it is, the CS and $ED_{i}$ successfully authenticate each other.

## 5. Security Weaknesses of Bhuarya et al.’s Scheme

In this section, we show that the scheme proposed by Bhuarya et al. does not prevent various potential attacks, such as impersonation and man-in-the-middle. Moreover, their scheme has an incorrect authentication mechanism and does not guarantee SMA, which is an essential requirement of an AKA protocol. This analysis was performed under the DY threat model described in Section 3.1.

### 5.1. Impersonation Attack

Owing to the fact that the scheme does not provide detailed tamper-proof techniques, we suppose that an adversary A obtains the embedded device $ED_{i}$ or captures it physically. Subsequently, A can access the data $\left\{PID_{i},R_{i},C_{k},I_{i}\right\}$ stored in $ED_{i}$ and perform impersonation attacks using the obtained data as follows:

(1) A chooses a random number $rn_{a}$ and a current timestamp $T_{1}$, and computes $P_{a}=rn_{a}\cdot G$, $P_{a2}=h\left(rn_{a}\cdot C_{k}^{\prime }\right)$, $E_{a}=PID_{i}⊕R_{i},K_{a}=h\left(P_{a}\right)⊕PID_{i}$, and $Y_{a}=h(P_{a}\left|\right|P_{a2}\left||K|\right|T_{1})$. Then, A sends the login request $\left\{E_{a},P_{a},Y_{a},T_{1}\right\}$ to CS.
(2) On receiving the login request from A, the CS checks the timestamp validity, computes $PID_{i}=E_{a}⊕R_{i}$, and finds $PID_{i}$ in the database.
(3) If it exists, the CS computes $rn_{s}=R_{i}⊕h(MX_{CS}||PID_{i})$, $C_{k}=h(rn_{s}||MX_{CS}\left|\right|E_{t}||PID_{i})$, $K=h(P_{a})$, $P_{a2}=h\left(P_{a}\cdot C_{k}\right)$, and $Y^{*}=h(P_{a}\left|\right|P_{a2}\left||K|\right|T_{1})$. The CS subsequently verifies that $Y^{*}$ is equal to *Y*. If it correct, the CS selects a random number $rn_{2}$ and a current timestamp $T_{3}$, and computes $P_{3}=rn_{2}\cdot G$, $P_{4}=rn_{2}\cdot A_{i}^{\prime }$, $S=h(P_{3}||P_{4}||T_{3})$. Afterwards, the CS sends the response messages $\left\{S,P_{3},T_{3}\right\}$ to A.
(4) After receiving $\left\{S,P_{3},T_{3}\right\}$ from CS, A computes $A_{a}=h\left(R_{i}⊕I_{i}⊕C_{k}^{\prime }\right)$, $P_{3}^{*}=P_{3}\cdot A_{a}$, and $S_{a}^{*}=h(P_{3}^{*}\left|\right|P_{4}\left|\right|T_{3})$, and then verifies that $S_{a}^{*}\overset{?}{=}S$ and timestamp is valid. If it is correct, A computes the session key $SK=h(rn_{a}\cdot P_{3}||PID_{i}||T_{4}||A_{a})$ and $V_{a}=h(\left(rn_{a}\cdot C_{k}^{\prime }\right)\left||SK\right)$, and then sends the messages $\left\{V_{a},T_{4}\right\}$ to CS.
(5) The CS checks the validity of the timestamp and computes the session key $SK^{*}=h(rn_{2}\cdot P_{a}||PID_{i}\left|\right|T_{4}\left|\right|A_{a})$ and $V_{a}^{*}=h((P_{a}\cdot C_{k}||SK^{*})$. Then, the CS verifies that $V_{a}^{*}$ is equal to $V_{a}$. If it is, the CS and A successfully authenticate each other.

A can successfully generate a valid login request $\left\{E_{a},P_{a},Y_{a},T_{1}\right\}$ and response messages $\left\{V_{a},T_{4}\right\}$, showing that the aforementioned scheme does not resist impersonation attacks.

### 5.2. Man-in-the-Middle Attack

An adversary A can perform a man-in-the-middle attack as follows:

(1) A first intercepts the login request $\left\{E,P_{1},Y,T_{1}\right\}$ of $ED_{i}$, and then chooses a random number $rn_{a}$ and a current timestamp $T_{1}$. A computes $P_{a}=rn_{a}\cdot G$, $P_{a2}=h\left(rn_{a}\cdot C_{k}^{\prime }\right)$, $E_{a}=PID_{i}⊕R_{i},K_{a}=h\left(P_{a}\right)⊕PID_{i}$, $Y_{a}=h(P_{a}\left|\right|P_{a2}\left||K|\right|T_{1})$, and sends the login request $\left\{E_{a},P_{a},Y_{a},T_{1}\right\}$ to CS.
(2) A chooses a random number $rn_{a2}$ and computes $P_{a3}=rn_{2}\cdot G$, $P_{a4}=rn_{2}\cdot A_{i}$, and $S_{a}=h(P_{a3}\left|\right|P_{a4}\left|\right|T_{3})$, where $A_{i}$ is obtained by the threat model.
(3) A intercepts the response messages $\left\{S,P_{3},T_{3}\right\}$ of the CS, and then computes $SK=h(rn_{a}\cdot P_{3}||PID_{i}||T_{4}||A_{a})$ and $V_{a}=h(\left(rn_{a}\cdot C_{k}^{\prime }\right)\left||SK\right)$. Finally, A sends $\left\{V_{a},T_{4}\right\}$ and $\left\{S_{a},P_{a3},T_{3}\right\}$ to the CS and $ED_{i}$, respectively.
(4) After receiving $\left\{V_{a},T_{4}\right\}$ and $\left\{S_{a},P_{a3},T_{3}\right\}$, the CS and $ED_{i}$ generates the session key using received messages.

A can successfully establish the session key SK using $rn_{a}$ and $rn_{a2}$, which shows that the aforementioned scheme does not prevent man-in-the-middle attacks.

### 5.3. Correctness of Authentication Mechanism

In the login and authentication phase of the scheme, the CS computes $\left\{S,P_{3},T_{3}\right\}$ and sends it to $ED_{i}$. Subsequently, $ED_{i}$ computes $S^{*}=h(P_{3}^{*}\left|\right|P_{4}\left|\right|T_{3})$ and verifies that $S\overset{?}{=}S^{*}$ to authenticate the CS. However, $ED_{i}$ cannot authenticate the CS because *S* is not equal to $S^{*}$ as follows:

$$\begin{array}{c} S=h(P_{3}\left|\right|P_{4}\left|\right|T_{3})=h(rn_{2}\cdot G||P_{4}\left|\right|T_{3})\\ S^{*}=h(P_{3}^{*}\left|\right|P_{4}\left|\right|T_{3})=h(P_{3}\cdot A_{i}\left|\right|P_{4}\left|\right|T_{3})\\ =h(rn_{2}\cdot G\cdot A_{i}||P_{4}||T_{3})\\ =h(rn_{2}\cdot G\cdot h(rn_{s}⊕h(MX_{cs}||PID_{i})⊕I_{i}⊕C_{k}^{\prime })||P_{4}\left|\right|T_{3})\\ ∴S\neq S^{*} \end{array}$$

### 5.4. Secure Mutual Authentication

In Section 5.1 and Section 5.2, we proved that the scheme proposed by Bhuarya et al. does not resist impersonation and man-in-the-middle attacks. Moreover, we proved that their scheme contains an incorrect authentication mechanism, which causes the authentication process to be aborted. Therefore, the scheme does not ensure SMA.

## 6. Proposed Scheme

This section presents an improved AKA scheme for IoT using PUF, which comprises three phases: system setup, registration, and login and authentication. In our scheme, embedded devices are tamper-proof devices that use a PUF to protect the data stored in memory. The embedded devices register their identities with the CS, authenticate them, and establish the session key to each other. After completing the AKA phase, $ED_{i}$ can use the various services offered by the CS.

### 6.1. System Setup Phase

The CS sets up the initial parameters related to the elliptic curve, which is identical to the Bhuarya et al. scheme. The CS then generates a secret key $MX_{CS}$ and broadcasts the initial public parameters.

### 6.2. Embedded Device Registration Phase

**Figure 1:**
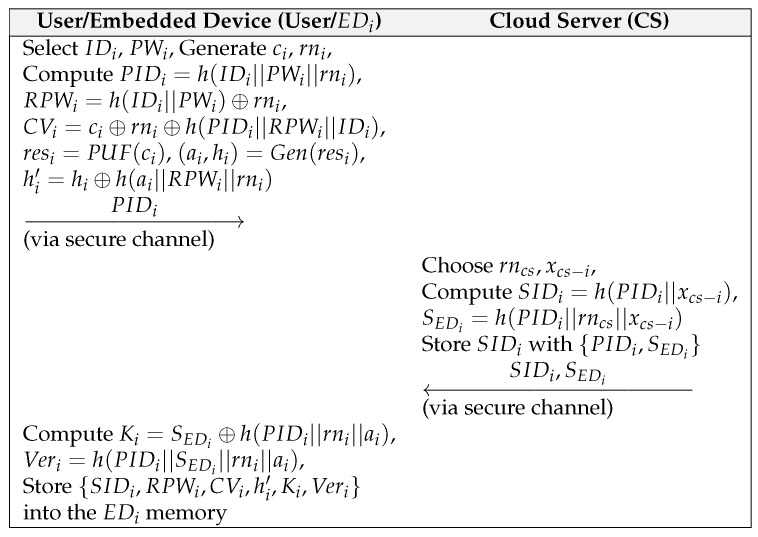
Registration Phase for Our Scheme.

This phase is shown in Figure 1, and the detailed steps are as follows:

(1) User selects identity $ID_{i}$, password $PW_{i}$, challenge $c_{i}$, and random number $rn_{i}$ for $ED_{i}$, and then computes $PID_{i}=h(ID_{i}||PW_{i}||rn_{i})$, $RPW_{i}=(ID_{i}||PW_{i})⊕rn_{i}$, and $CV_{i}=c_{i}⊕rn_{i}⊕h(PID_{i}||RPW_{i}||ID_{i})$. $ED_{i}$ calculates $res_{i}=PUF\left(c_{i}\right)$ and $\left(a_{i},h_{i}\right)=Gen\left(res_{i}\right)$ using the PUF and fuzzy extractor. Afterwards, $ED_{i}$ computes $h_{i}^{\prime }=h_{i}⊕h(a_{i}||RPW_{i}||rn_{i})$ and sends $\left\{PID_{i}\right\}$ to the CS via a secure channel.
(2) On receiving the registration request from $ED_{i}$, the CS chooses a random number $x_{cs-ED_{i}}$ for $ED_{i}$, and then computes $SID_{i}=h(PID_{i}\left|\right|S_{cs-ED_{i}}$ and $S_{ED_{i}}=h(PID_{i}||rn_{cs}\left|\right|x_{cs-ED_{i}})$. The CS stores $SID_{i}$ with $\left\{PID_{i},S_{ED_{i}}\right\}$ in a secure database and sends $\left\{SID_{i},S_{ED_{i}}\right\}$ to $ED_{i}$ through a secure channel.
(3) After receiving $\left\{SID_{i},S_{ED_{i}}\right\}$ from the CS, $ED_{i}$ computes $K_{i}=S_{ED_{i}}⊕h(PID_{i}||rn_{i}\left|\right|a_{i})$ and $Ver_{i}=h(PID_{i}\left|\right|S_{ED_{i}}||rn_{i}\left|\right|a_{i})$, and stores $\left\{SID_{i},RPW_{i},CV_{i},h_{i}^{\prime },K_{i},Ver_{i}\right\}$ in memory.

### 6.3. Authentication and Key Agreement Phase

**Figure 2:**
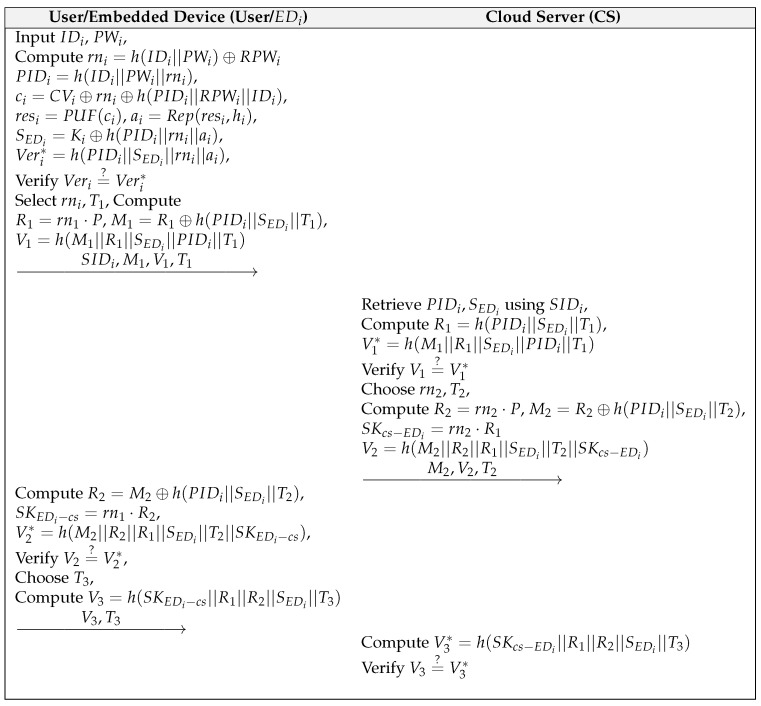
Login and Authentication Phase for Our Scheme.

This phase is shown in Figure 2, and the detailed steps are as follows:

(1) User inputs the identity $ID_{i}$ with password $PW_{i}$ to $ED_{i}$, and then $ED_{i}$ computes $h_{ID||PW_{i}}$, $rn_{i}=h_{ID||PW_{i}}⊕RPW_{i}$, $PID_{i}=h(ID_{i}||PW_{i}||rn_{i})$, $c_{i}=CV_{i}⊕rn_{i}⊕h(PID_{i}||RPW_{i}||ID_{i})$, $res_{i}=PUF\left(c_{i}\right)$, $h_{i}=h_{i}^{\prime }⊕h(c_{i}||PID_{i}||ID_{i})$, $a_{i}=Rep\left(res_{i},h_{i}\right)$, $S_{ED_{i}}=K_{i}⊕h(PID_{i}||rn_{i}\left|\right|a_{i})$ and $Ver_{i}^{*}=h(PID_{i}\left|\right|S_{ED_{i}}||rn_{i}\left|\right|a_{i})$. $ED_{i}$ checks whether $Ver_{i}^{*}\overset{?}{=}Ver_{i}$. If it is correct, $ED_{i}$ chooses a random number $rn_{1}$ and a current timestamp $T_{1}$; otherwise, it aborts the connection. $ED_{i}$ computes $R_{1}=rn_{1}\cdot P$, $M_{1}=R_{1}⊕h(PID_{i}\left|\right|S_{ED_{i}}\left|\right|T_{1})$, and $V_{1}=h(M_{1}\left|\right|R_{1}\left|\right|S_{ED_{i}}||PID_{i}||ID_{cs}\left|\right|T_{1})$, and then sends $\left\{SID_{i},M_{1},V_{1},T_{1}\right\}$ to the CS.
(2) On receiving the login request from $ED_{i}$, the CS checks the timestamp validity and finds $\left\{PID_{i},S_{ED_{i}}\right\}$ using $SID_{i}$ from a secure database. The CS computes $h(PID_{i}||S_{ED_{i}}||T_{1})$, $R_{1}=M_{1}⊕h(PID_{i}\left|\right|S_{ED_{i}}\left|\right|T_{1})$ and $V_{1}^{*}=h(M_{1}\left|\right|R_{1}\left|\right|S_{ED_{i}}||PID_{i}||ID_{cs}\left|\right|T_{1})$, and then verifies that $V_{1}^{*}$ is equal to $V_{1}$.
(3) If it is equal, the CS generates computes a random number $rn_{2}$ and a current timestamp $T_{2}$; otherwise, aborts the connection. The CS calculates $R_{2}=rn_{2}\cdot P$, $M_{2}=R_{2}⊕h(PID_{i}\left|\right|S_{ED_{i}}\left|\right|T_{2})$, the session key $SK_{cs-ED_{i}}=rn_{2}\cdot R_{1}$, and $V_{2}=h(M_{2}\left|\right|R_{2}\left|\right|R_{1}\left|\right|S_{ED_{i}}||ID_{cs}||SK_{cs-ED_{i}})$. After that, the CS sends the response messages $\left\{M_{2},V_{2},T_{2}\right\}$ to $ED_{i}$.
(4) After receiving $\left\{M_{2},V_{2},T_{2}\right\}$ from the CS, $ED_{i}$ checks timestamp validity and computes $h(PID_{i}||S_{ED_{i}}||T_{2})$, $R_{2}=M_{2}⊕h(PID_{i}\left|\right|S_{ED_{i}}\left|\right|T_{2})$, the session key $SK_{ED_{i}-cs}=rn_{1}\cdot R_{2}$, and $V_{2}^{*}=h(M_{2}\left|\right|R_{2}\left|\right|R_{1}\left|\right|S_{ED_{i}}||ID_{cs}||SK_{ED_{i}-cs})$. Then, $ED_{i}$ checks whether $V_{2}^{*}\overset{?}{=}V_{2}$. If it is verified, $ED_{i}$ generates a current timestamp $T_{3}$ and computes $V_{3}=h(SK_{ED_{i}-cs}\left|\right|R_{1}\left|\right|R_{2}\left|\right|S_{ED_{i}}\left|\right|T_{3}$. $ED_{i}$ sends the verification messages $\left\{V_{3},T_{3}\right\}$ to the CS.
(5) On receiving $\left\{V_{3},T_{3}\right\}$ to $ED_{i}$, the CS computes $V_{3}^{*}=h(SK_{cs-ED_{i}}\left|\right|R_{1}\left|\right|R_{2}\left|\right|S_{ED_{i}}\left|\right|T_{3}$ and checks its validity. If it is verified, the CS and $ED_{i}$ successfully authenticate each other.

## 7. Security Analysis

In this section, we prove that iAKA-CIoT ensures the session key security (SKS) using the real-or-random (RoR) model [25]. We also perform an informal analysis and simulation analysis using the AVISPA verification tool [18] to demonstrate that our scheme is secure against various potential attacks.

### 7.1. Formal Security Analysis Using ROR Model

We prove that our scheme achieves SKS using an ROR model-based mathematical formal proof [26,27,28]. We first discuss the fundamental concept and queries of the ROR model before conducting the formal analysis.

- Participants: Let $\Pi_{ED}^{inst_{1}}$ and $\Pi_{CS}^{inst_{2}}$ be the instance $inst_{1}$ and $inst_{2}$ of the ED and CS, respectively.
- Accepted state: After completing the message exchanging process, the oracle $\Pi^{i}nst$ transfers a this state. Let the current session identifier be $sid_{c}$ of $\Pi^{inst}$ should all the messages be arranged in order.
- Partnering: When $\Pi_{ED}^{inst_{1}}$ and $\Pi_{CS}^{inst_{2}}$ have the same $sid_{c}$ and the accepted state, and each oracle completes the AKA, partners ($\Pi_{ED}^{inst_{1}}$ and $\Pi_{CS}^{inst_{2}}$) are defined.
- Freshness: To carry out the formal proof, $\Pi_{ED}^{inst_{1}}$ and $\Pi_{CS}^{inst_{2}}$ as instances are deemed fresh if the session key between the ED and CS is presently not revealed to adversary *A*.
- Attacker: Under our enhanced threat model Section 3.1, *A* can completely control the public network and send the ROR queries shown in Table 2 to destroy the SKS.
- Semantic Security: *A* tries to find a correct session key from a random number utilizing the ROR queries. If *A* correctly guesses a bit *c*, *A* wins this game and breaks the semantic security of the scheme. Let $Adv_{P}=\left|2Pr\left[Succ\right]-1\right|$ be the advantage in breaking the session key of scheme P, where Win is the event of the winning game by *A*.
- Random oracle: All participant entities can use a random oracle as a collision resistant one-way hash function Hash.

Now, we prove that our scheme ensures SKS using the following Definitions 1 and 2 and Theorem 1.

**Definition** **1.** Elliptic curve discrete logarithm problem (ECDLP): Given P and Q, it is computationally intractable to find integer a such that Q=a·P, where $a\in Z_{p}^{*}$.

**Definition** **2.** Elliptic curve decision Diffie–Hellman problem (ECDDHP): Given P,xP, and yP, it is computationally difficult to compute x·y·P, where $x,y\in Z_{p}^{*}$.

**Theorem** **1.** *Let an adversary run in polynomial time t as A, and let the advantage of A in breaking the SKS be $Adv_{P}^{A}$. Then,*

(1) $$\begin{array}{c} Adv_{P}^{A}\leq \frac{q_{h}^{2}}{2|Hash|}+\frac{q_{puf}^{2}}{2|PUF|}+max\left\{C^{\prime },q_{s}^{s^{\prime }},\frac{q_{s}}{2^{lenf}},\frac{q_{s}}{2^{lenp}}\right\} \end{array}$$

*where $q_{h}$, Hash, and $Adv^{ECDLP}\left(t\right)$ is the number of Hash queries, a collision-resistant hash function Hash, and an advantage in breaking ECDLP, respectively.*

The formal proofs consisting of four games $G_{i}\left(i=0,1,2\right)$ using the ROR model are as follows:

- Game $G_{0}$: *A* first tosses the coin *c* and obtains its result at the beginning of this game. Its winning advantage is:
  (2) $$Adv_{P}^{A}=\left|2.Pr\left[Succ_{0}\right]-1\right|,$$
  where Succ is the event of *A* winning the game.
- Game $G_{1}$: Under this game, Attacker *A* performs an eavesdropping attack using the $Execute(\Pi_{ED}^{inst_{1}},$$\Pi_{CS}^{inst_{2}})$ query. *A* first intercepts the transmitted messages $\left\{SID_{i},M_{1},V_{1},T_{1}\right\}$, $\left\{M_{2},V_{2},T_{2}\right\}$, and $\left\{V_{3},T_{3}\right\}$ to break the SKS. Then, *A* executes the $Test(\Pi^{t})$ query to guess whether the output of the query is equal to SK or any arbitrary number. However, the winning probability of $G_{1}$ does not increase because *A* does not compute the session key $SK_{ED_{i}-cs}=rn_{1}\cdot rn_{2}\cdot P$ without breaking the ECDLP and ECDDHP. Thus, we obtain:
  (3) $$Pr\left[Succ_{1}\right]=Pr\left[Succ_{0}\right]$$
- Game $G_{2}$: Attacker *A* performs an active attack using $Send(\Pi^{inst},M)$ and Hash queries. *A* attempts to guess the correct message digest collision to mislead a participant entity using several Hash queries. However, in our scheme, all transmitted messages are secured because *A* does not break the Hash oracle in polynomial time. Moreover, *A* cannot compute the correct messages without the pseudo-identity $PID_{i}$, secret value $S_{ED_{i}}$, and tamper-proof value $a_{i}$. Thus, according to the birthday paradox [29],
  (4) $$|Pr\left[Succ_{1}\right]-Pr\left[Succ_{2}\right|\leq \frac{q_{h}^{2}}{2|Hash|}$$
- Game $G_{3}$: Attacker *A* performs a final attack and can obtain $\left\{SID_{i},RPW_{i},CV_{i},h_{i}^{\prime },K_{i},Ver_{i}\right\}$ stored in the memory of $ED_{i}$ using $CorruptED(\Pi_{ED}^{inst_{1}})$. However, *A* does not compute the valid login request messages $\left\{SID_{i},M_{1},V_{1},T_{1}\right\}$ without knowing $\left\{ID_{i},PW_{i},a_{i}\right\}$, where $M_{1}=R_{1}⊕h(PID_{i}\left|\right|S_{ED_{i}}\left|\right|T_{1})$ and $V_{1}=h(M_{1}\left|\right|R_{1}\left|\right|S_{ED_{i}}||PID_{i}||ID_{cs}\left|\right|T_{1})$. Since *A* does not know $ID_{i}$, $rn_{i}$, $PID_{i}$ and $a_{i}$, *A* cannot correctly guess $PW_{i}$ using $Send(\Pi^{inst},M)$. Moreover, $a_{i}$ is only generated by the secure PUF function with a fuzzy extractor, which is defined in Section 3.2, and *A* does not distinguish between the PUF values and those of the noise without help of fuzzy extractor because the guessing probability of fuzzy extractor values lenf and lenp is approximately $\frac{1}{2^{lenf}}$ and $\frac{1}{2^{lenp}}$, respectively. Therefore, from the PUF simulation and Zipf’s law on passwords [30],
  (5) $$\begin{array}{c} |Pr\left[Succ_{1}\right]-Pr\left[Succ_{2}\right|\leq \frac{q_{puf}^{2}}{2|PUF|}+max\left\{C^{\prime },q_{s}^{s^{\prime }},\frac{q_{s}}{2^{lenf}},\frac{q_{s}}{2^{lenp}}\right\} \end{array}$$

After simulating all the games ($G_{0},G_{1},G_{2},G_{3})$, *A* attempts to guess the correct *c* using the Test query. Therefore,

(6) $$\begin{array}{c} Adv_{P,G_{3}}^{A}=\frac{1}{2} \end{array}$$

We can obtain the following results using Equations (2), (3) and (6).

(7) $$\begin{array}{ccc} \frac{1}{2}.Adv_{P}^{A} & = & |Pr\left[Succ_{0}\right]-\frac{1}{2}|\\ & = & |Pr\left[Succ_{1}\right]-\frac{1}{2}|\\ & = & |Pr\left[Succ_{1}\right]-Pr\left[Succ_{3}\right]| \end{array}$$

Then, we can gain the following results using (5)–(7):

(8) $$\begin{array}{ccc} \frac{1}{2}.Adv_{P}^{A} & = & |Pr\left[Succ_{1}\right]-Pr\left[Succ_{3}\right]|\\ & \leq & |Pr\left[Succ_{1}\right]-Pr\left[Succ_{2}\right]|\\ & + & |Pr\left[Succ_{2}\right]-Pr\left[Succ_{3}\right]|\\ & \leq & \frac{q_{h}^{2}}{2|Hash|}+\frac{q_{puf}^{2}}{2|PUF|}+max\left\{C^{\prime },q_{s}^{s^{\prime }},\frac{q_{s}}{2^{lenf}},\frac{q_{s}}{2^{lenp}}\right\} \end{array}$$

Finally, we acquire the final goal by multiplying both sides of (8) by two.

(9) $$\begin{array}{c} Adv_{P}^{A}\leq \frac{q_{h}^{2}}{2|Hash|}+\frac{q_{puf}^{2}}{2|PUF|}+max\left\{C^{\prime },q_{s}^{s^{\prime }},\frac{q_{s}}{2^{lenf}},\frac{q_{s}}{2^{lenp}}\right\} \end{array}$$

### 7.2. Informal Security Analysis

This section demonstrates that our scheme is secure against various potential attacks, such as impersonation, man-in-the-middle, replay, physical capture, and offline password guessing. In addition, we demonstrate that it guarantees SMA and anonymity.

#### 7.2.1. Impersonation Attack

Under our threat model, an adversary A can acquire the exchanged messages in a public network and extract the stored data $\left\{SID_{i},RPW_{i},CV_{i},h_{i}^{\prime },K_{i},Ver_{i}\right\}$ from the memory of $ED_{i}$. However, A cannot attempt to impersonate a legitimate $ED_{i}$ because A does not successfully generate the login request $\left\{SID_{i},M_{1},V_{1},T_{1}\right\}$ and verification messages $\left\{V_{3},T_{3}\right\}$ without knowing $ID_{i}$, $PW_{i}$, $S_{ED_{i}}$ and $a_{i}$. Therefore, iAKA-CIoT is secure against impersonation attacks.

#### 7.2.2. Man-in-the-Middle Attack and Replay Attack

When A tries to perform a man-in-the-middle attack, A should obtain $\left\{R_{1},R_{2}\right\}$ and compute the response messages $\left\{M_{2},V_{2}\right\}$ and $\left\{V_{3}\right\}$. However, A cannot obtain $R_{1}$ and $R_{2}$ without obtaining $h(PID_{i}||rn_{i}||a_{i})$. Moreover, all response messages include a timestamp and are masked by a collision-resistant hash function, which makes it difficult to find original messages in polynomial time. Therefore, iAKA-CIoT resists man-in-the-middle and replay attacks.

#### 7.2.3. Physical Capture Attack

After obtaining the data $\left\{SID_{i},RPW_{i},CV_{i},h_{i}^{\prime },K_{i},Ver_{i}\right\}$ stored in the memory of $ED_{i}$’ using a physical capture attack, the data do not help compute the session key SK because the PUF response $a_{i}$ is only generated by $ED_{i}$ and A cannot retrieve $S_{ED_{i}}$ from $K_{i}$. Therefore, our scheme protects against physical-capture attacks.

#### 7.2.4. Offline Password Guessing Attack

We assume that A attempts to guess the password of the user by using intercepted messages and extracting data. A must know the real identity $ID_{i}$, random number $rn_{i}$, pseudo identity $PID_{i}$ and secure parameter $S_{ED_{i}}$. However, A does not know these values because it is masked by a collision-resistant hash function, and A cannot simultaneously guess two or three parameters in polynomial time. Therefore, iAKA-CIoT is secure against offline password-guessing attacks.

#### 7.2.5. Secure Mutual Authentication and Anonymity

In the AKA phase of our scheme, the CS and $ED_{i}$ verify the login request $V_{1}\overset{?}{=}V_{1}^{*}$ and response messages $V_{2}\overset{?}{=}V_{2}^{*}$ by using $PID_{i}$ and $S_{ED_{i}}$. According to previous analyses (Section 7.2.1, Section 7.2.2 and Section 7.2.3), A does not compute verification messages $V_{1}$ and $V_{2}$ without obtaining $\left\{PID_{i},a_{i},ID_{i},PW_{i},S_{ED_{i}}\right\}$. Moreover, in our scheme, the user utilizes the pseudo identity $PID_{i}$ for the AKA phase, and A cannot obtain the real identity $ID_{i}$ of the user. Therefore, our scheme achieves SMA and anonymity.

#### 7.2.6. Denial-of-Service Attack

After receiving exchanged messages between CS and $ED_{i}$, they should perform verification procedures to prove validity of these messages {$Ver_{i},V_{1},V_{3}$}. If it is not valid, the AKA procedure is immediately aborted. It can mitigate denial of service (DoS)/distributed denial of service (DDoS) attacks because {$Ver_{i},V_{1},V_{3}$} has freshness which includes timestamp and random number, and can be generated by a legitimate entities.

### 7.3. Simulation Analysis Using AVISPA Tool

**Figure 3:**
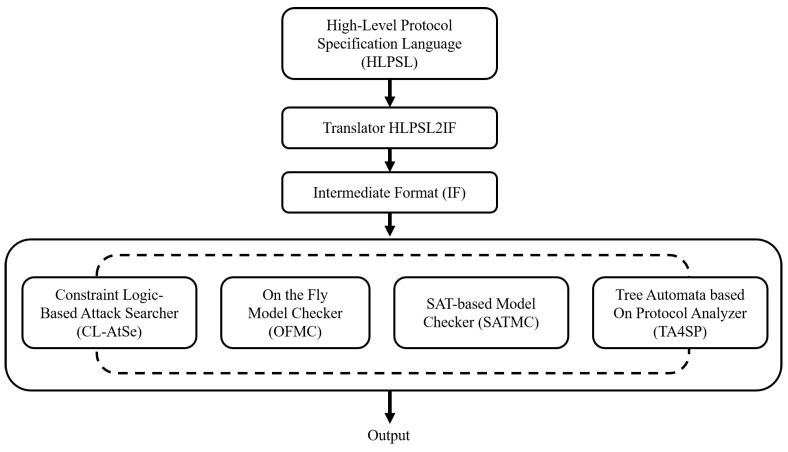
The Procedures of AVISPA Simulation.

In this section, we discuss the simulation of our scheme by using the AVISPA simulation tool to prove its security [18,31]. AVISPA is a well-known formal simulation tool for evaluating the security of protocols, whereby it verifies that a protocol resists man-in-the-middle and replay attacks. First, we define the security properties of our scheme by using a high-level protocol specification language (HLPSL) [32]. The defined HLPSL code was transformed into an intermediate format using the HLPSL2IF translator. This simulation was executed under the four back-ends model [33]; “on-the-fly model checker” (OFMC); “tree automata based on a protocol analyzer” (TA4SP); “SAT-based model checker” (SATMC), and “constraint logic-based attack searcher” (CL-AtSE). The procedure of this simulation is shown in Figure 3 and the concept of HLPSL is presented in [31,32].

#### 7.3.1. HLPSL Specifications

**Figure 4:**
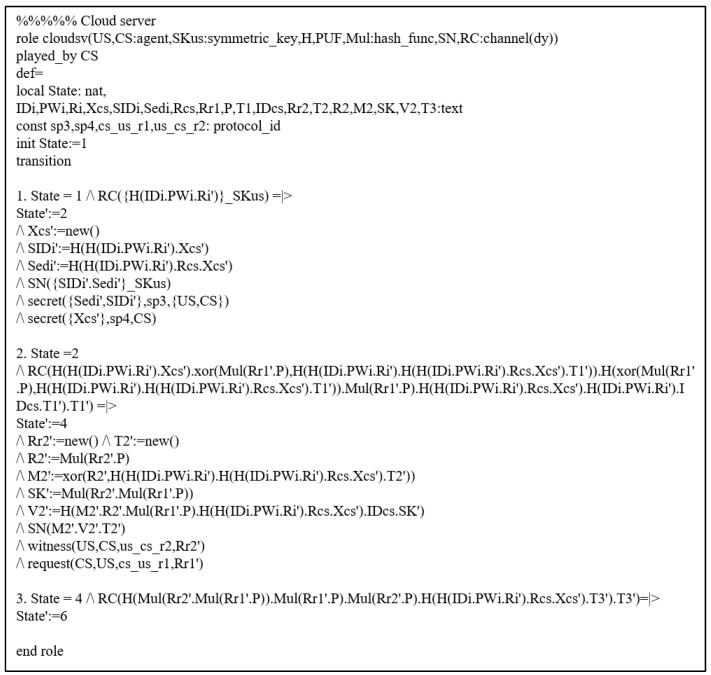
HLPSL Description: CS Role.

**Figure 5:**
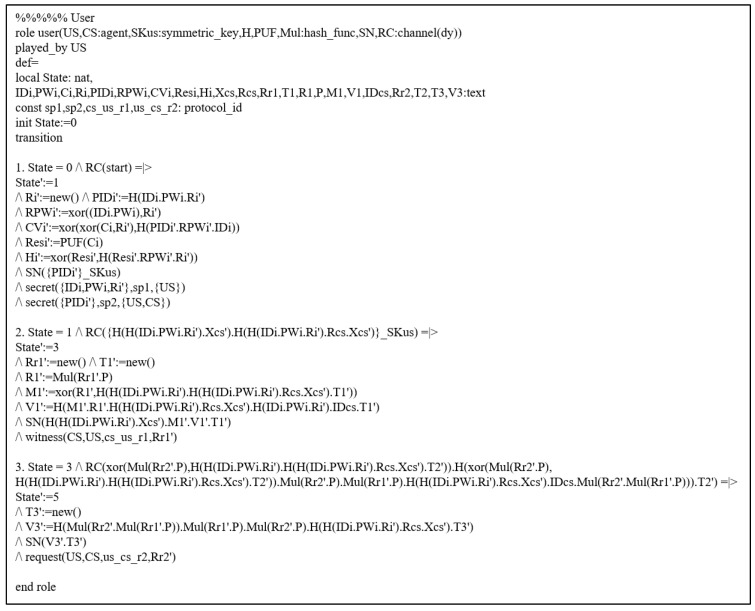
HLPSL Description: $ED_{i}$ role.

**Figure 6:**
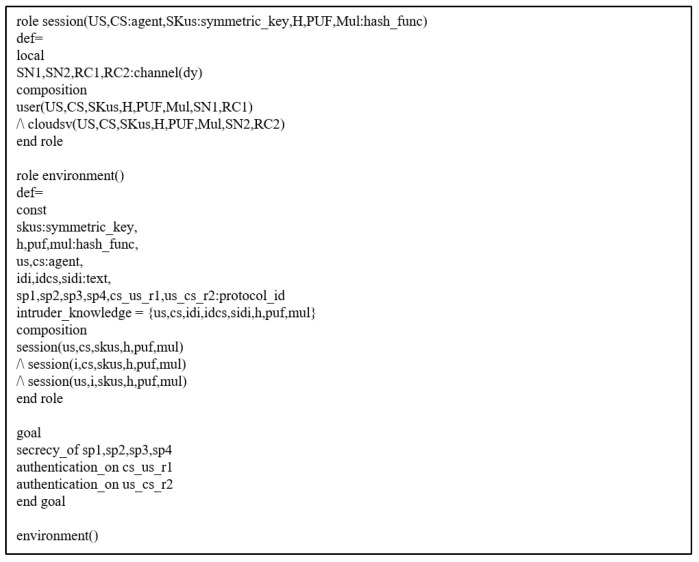
HLPSL Description: Session and Environment.

We simulated the defined HLPSL by considering the $ED_{i}$ and CS AKA phase. There are two basic roles (CS, $ED_{i}$), and their HLPSL descriptions are presented in Figure 4 and Figure 5. A session with the environment is defined in Figure 6.

#### 7.3.2. Simulation Results

**Figure 7:**
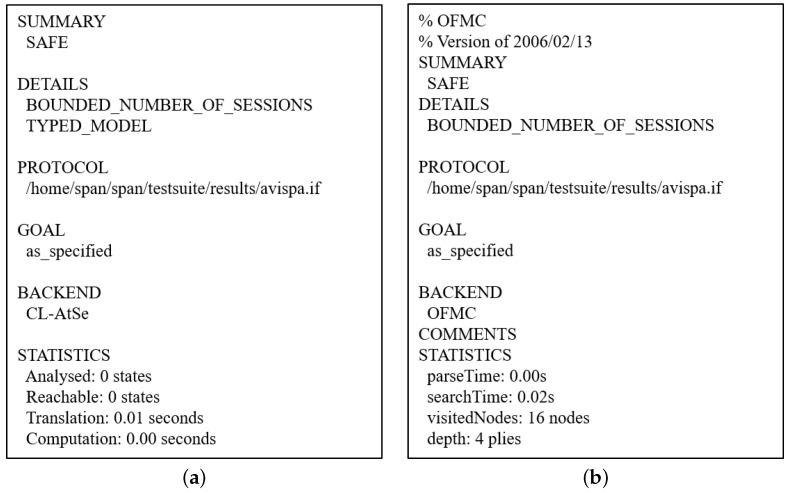
Simulation Result of AVISP Simulation (Summary: SAFE). (**a**) Result of CL-AtSe. (**b**) Result of OFMC.

Figure 7 shows the results of the AVISPA simulation, which presents the simulation summary “SAFE”. In the CL-AtSe results, the translation time was 0.01 s. For the OFMC results, the search depth was four when 16 nodes were explored in 0.02 s. Therefore, our scheme prevents man-in-the-middle and replay attacks.

## 8. Comparative Analysis

This section presents a comparative analysis of our scheme on the security property, communication, and computation cost with other related schemes [6,10,11,16,17].

### 8.1. Security Property

We compared the security properties of our scheme with those of the contemporary schemes. Table 3 shows that the previous schemes cannot resist security attacks, achieve anonymity, or SMA. In contrast, we demonstrate that iAKA-CIoT can prevent potential security attacks and guarantee essential security requirements. Therefore, our scheme is more secure than the aforementioned schemes [6,10,11,13,14,16,17].

We demonstrated that Bhuarya et al. [11] is insecure against physical capture attacks in Section 5. We also proved that other related schemes [6,10,16] does not prevent physical capture attacks to highlight our contributions. The detailed processes of AKA schemes refer to [6,10,16,17].

In [6], an adversary A can extract the data {$Pid_{i},C_{k}^{\prime }$} stored in the embedded device $ED_{i}$, and then A selects a random number and computes $P_{1}=r_{1}\cdot G$, $P_{2}=h(r_{1}\cdot C_{k}^{\prime })$. Finally A can successfully generate the login request messages $\left\{P_{1},P_{2},Pid_{i}\right\}$ without knowing any other information.

In [10], A can obtain the parameters $\left\{B_{i},N_{i},N_{t}\right\}$ and $\left\{W_{i},V_{i},T_{u}\right\}$ from the user’s smart card and open channel. Then, A tries to obtain the $PW_{i}$ by executing offline password guessing attacks [34]. A chooses {$ID_{i}^{*},PW_{i}^{*}\}$, and computes $k^{*}=N_{i}⊕h\left(ID_{i}^{*}⊕PW_{i}^{*}\right)$, $A_{i}^{*}=B_{i}⊕h(ID_{i}^{*}||h(PW_{i}^{*}\left|\right|k^{*})$, ${\left(r_{i}⊕k\right)}^{*}=W_{i}⊕h(T_{u}⊕A_{i}^{*}$) and $V_{i}^{*}=h(ID_{i}^{*}\left|\right|A_{i}^{*}\left|\right|W_{i}\left|\right|{\left(r_{i}⊕k\right)}^{*}\left|\right|T_{u}$. If $V_{i}$ is equal to $V_{i}^{*}$, A successfully guesses the correct $PW_{i}$ and can correctly generate valid login request.

In [16], we assumed that A is a dishonest registered participant in the system. Then, A can extract the data $\left\{AID_{A},BID_{A},r_{A}\right\}$ from smart card and can impersonate a legitimate user $U_{a}$ using it. In their scheme, A can establish the session key of any legitimate user by betraying a trusted server [7].

Therefore, the aforementioned schemes are insecure against physical capture attacks because they stored secret data as plaintext, which causes critical security issues.

### 8.2. Computation, Communication and Storage Costs

**Figure 8:**
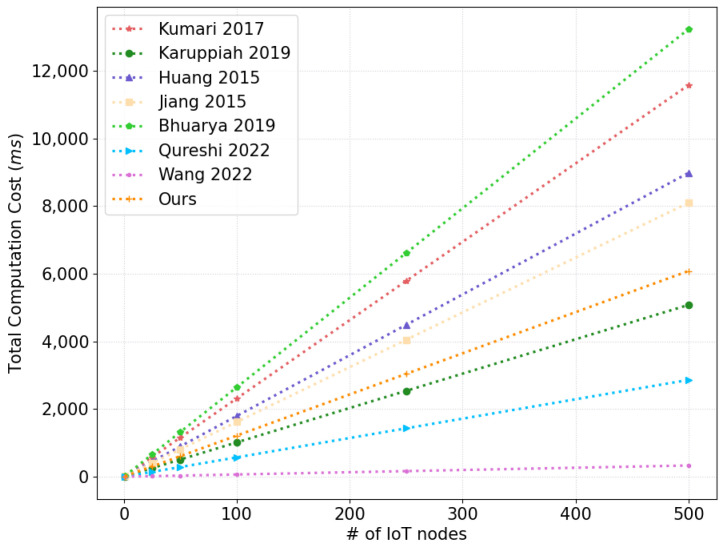
A Comparative Analysis: Computational Cost (Figure).

**Figure 9:**
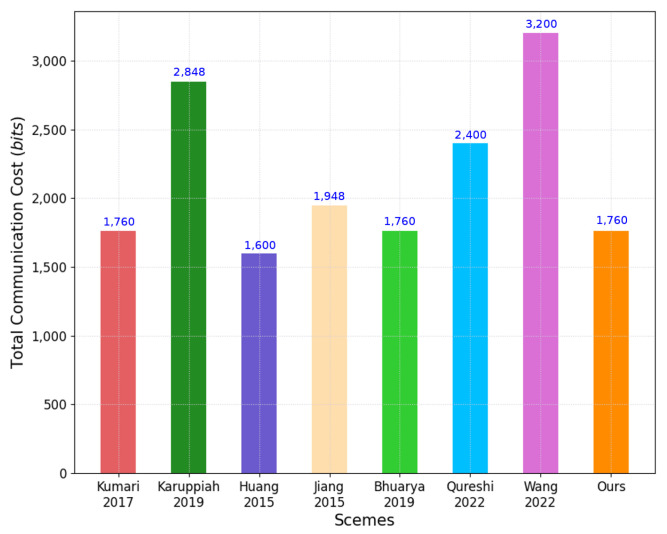
A Comparative Analysis: Communication Cost (Figure).

**Figure 10:**
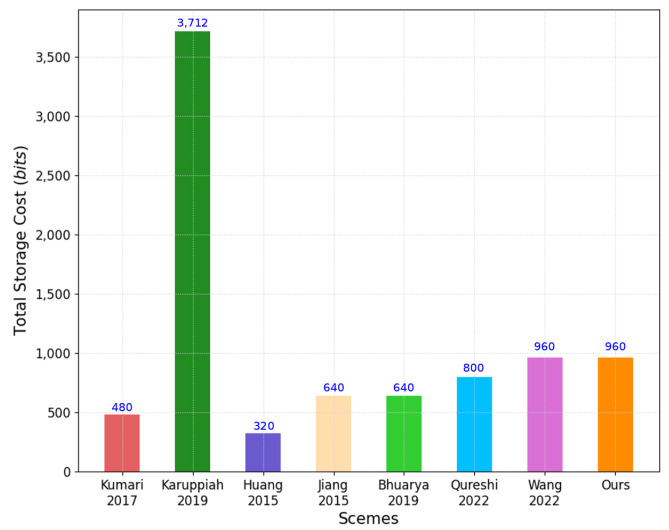
A Comparative Analysis: Storage Cost (Figure).

In this analysis, we consider the AKA phase for protocols. Table 4, Table 5 and Table 6 compare the computation, communication and storage costs between our scheme and other related schemes, which is shown in Figure 8, Figure 9 and Figure 10, respectively.

The computation cost analysis was performed using Raspberry PI 4B with Linux Ubuntu 18.04.4 LTS with 64-bits, 8 GB, and MIRACL library. We utilized the average values for each cryptographic primitive, which was run 100 times to measure its execution cost. To evaluate the computational cost of iAKA-CIoT compared with other schemes, we considered four cryptographic primitives, and their execution costs are presented in Table 7.

Our scheme requires the total cost $15T_{h}+4T_{em}\approx 0.765+11.392=12.157$ ms, whereas the total cost for other schemes are as follows: that in Kumari et al. [6] required $7T_{h}+8T_{em}\approx 0.357+22.784=23.141$ ms; that in Karuppiah et al. [10] required 0.714+9.438=10.152 ms; that in Huang et al. [16] required $17T_{h}+6T_{em}\approx 0.867+17.088=17.955$ ms; that in Jiang et al. [17] required $9T_{h}+5T_{modexp}\approx 0.459+15.73=16.189$ ms; that in Bhuarya et al. [11] required $16T_{h}+9T_{em}\approx 0.816+25.632=26.448$ ms; that in Qureshi and Munir [13] required $2T_{aes}+2T_{em}\approx 0.024+5.696=5.72$ ms; that in Wang et al. [14] required $13T_{h}\approx 0.663$ ms.

For the comparison of communication costs, we defined the message length of the parameters. The one-way hash function, identity, timestamp, and random number are 160 bits. The elliptic curve point and modular exponentiation are 320 and 1024 bits, respectively. In our scheme, the exchanged messages $\left\{SID_{i},M_{1},V_{1},T_{1}\right\}$, $\left\{M_{2},V_{2},T_{2}\right\}$, and $\left\{V_{3},T_{3}\right\}$ needs 160+320+160+160=800 bits, 320+160+160=640 bits, and 160+160=320 bits, respectively. Thus, the total communication cost for our scheme was 800+640+320=1760. Kumari et al. [6], Karuppiah et al. [10], Huang et al. [16], Jiang et al. [17], Bhuarya et al. [11], Qureshi and Munir [13] and Wang et al. [14] required 1760, 2848, 1600, 1984, 1760, 2400, and 3200, respectively.

The iAKA-CIoT requires a storage cost of 960 bits, whereas the storage cost for other schemes are as follows: that in Kumari et al. [6] required 480 bits; that in Karuppiah et al. [10] required 3712 bits; that in Huang et al. [16] required 320 bits; that in Jiang et al. [17] required 640 bits; that in Bhuarya et al. [11] required 640 bits; that in Qureshi and Munir [13] required 800 bits; and that in Wang et al. [14] required 960 bits.

Section 8.1 shows that the abovementioned schemes [6,10,11,13,14,16,17] are insecure against various attacks such as password guessing, impersonation, replay, and physical capture attacks. Moreover, their schemes do not provide anonymity, a formal proof analysis, or SMA. Although some schemes [13,14] can prevent physical capture attacks, their scheme has security weaknesses [15] or high communication costs. Therefore, our scheme is secure and superior for practical IoT environments.

## 9. Conclusions

This paper demonstrated that the Bhuarya et al. scheme had an incorrect authentication mechanism, did not resist various attacks, such as impersonation, man-in-the-middle, and physical capture attacks. We also demonstrated that it did not achieve SMA and SKS. We proposed an improved authentication and key agreement scheme for cloud-enabled IoT using PUF to resolve these security flaws. We demonstrated that iAKA-CIoT is secure against impersonation, man-in-the-middle, replay, offline-password guessing, and physical capture attacks, and achieves SMA and anonymity. Formal security proof confirmed that our scheme achieved SKS between the CS and ED using the ROR model. Moreover, we performed a formal simulation analysis using the AVISPA tool and compared it with other related schemes using the Raspberry PI 4B with MIRACL library. Our scheme also provides superior security properties compared to the aforementioned schemes. Therefore, iAKA-CIoT is suitable for practical cloud-enabled IoT environments because it is more secure and superior than the other related schemes.

## Figures and Tables

**Table 1:** Notations.

Notation	Description
$ED_{i}$	Embedded device
CS	Cloud server
$ID_{i}$	Identity of $ED_{i}$
$PID_{i}$	Pseudo identity of $ED_{i}$
$ID_{CS}$	Identity of the CS
$MX_{CS}$	Master secret key of the CS
$x_{cs-i}$	A shared secret value between the CS and $ED_{i}$
$SK_{cs-ED_{i}}$	A session key between the CS and $ED_{i}$
$rn_{i}$	Random number chosen by entities
⊕	Bit-wise XOR function
h(·)	One-way hash function
||	A concatenation operation

**Table 2:** Queries with their descriptions.

Queries	Descriptions
$Execute(\Pi_{ED}^{inst_{1}},$ $\Pi_{CS}^{inst_{2}})$	*A* can perform an eavesdropping attack using this query under the threat model
$CorruptED(\Pi_{ED}^{inst_{1}})$	*A* can perform device stolen attacks using it to retrieve the data stored in $ED_{i}$.
$Send(\Pi^{inst},M)$	*A* can send messages and receive its response from the oracle $P^{inst}$ using it.
$Test(\Pi^{inst})$	Under this query, *A* guesses the probabilistic result for an unbiased coin *c*. When the freshness of the session key SK is established by $P^{inst}$ and *A*, *A* guesses SK by sending a Test query to the oracle. If c=c or c=1, *A* obtain an arbitrary number or the correct SK, respectively; otherwise, obtains the NULL (⊥).

**Table 3:** A Comparative Summary: Security Properties.

Properties	[6]	[10]	[16]	[17]	[11]	[13]	[14]	Ours
$SP_{1}$	×	×	×	×	×	*√*	*√*	*√*
$SP_{2}$	×	×	×	×	×	*√*	×	*√*
$SP_{3}$	*√*	×	×	*√*	*√*	N/A	*√*	*√*
$SP_{4}$	×	*√*	*√*	*√*	*√*	*√*	*√*	*√*
$SP_{5}$	×	×	×	×	×	*√*	×	*√*
$SP_{6}$	*√*	*√*	*√*	×	*√*	×	×	*√*
$SP_{7}$	×	*√*	×	×	×	*√*	×	*√*
$SP_{8}$	*√*	×	×	×	*√*	×	*√*	*√*

*√*: supports the security property; ×: does not support the security property; N/A: not applicable; $SP_{1}$: physical capture attack; $SP_{2}$: impersonation attack; $SP_{3}$: offline password guessing attack; $SP_{4}$: replay attack; $SP_{5}$: mutual authentication; $SP_{6}$: user anonymity; $SP_{7}$: formal (mathematical) proof; $SP_{8}$: formal simulation proof.

**Table 4:** A Comparative Analysis: Computational Cost.

Scheme	Login Procedure	Authentication Procedure	Total Costs
Kumari et al. [6]	$4T_{h}+4T_{em}$	$3T_{h}+4T_{em}$	$7T_{h}+8T_{em}\approx 0.357+22.784=23.141$ ms
Karuppiah et al. [10]	$10T_{h}+3T_{modexp}$	$4T_{h}$	$14T_{h}+3T_{modexp}\approx 0.714+9.438=10.152$ ms
Huang et al. [16]	$12T_{h}+5T_{em}$	$5T_{h}+1T_{em}$	$17T_{h}+6T_{em}\approx 0.867+17.088=17.955$ ms
Jiang et al. [17]	$6T_{h}+5T_{modexp}$	$3T_{h}$	$9T_{h}+5T_{modexp}\approx 0.459+15.73=16.189$ ms
Bhuarya et al. [11]	$10T_{h}+5T_{em}$	$6T_{h}+4T_{em}$	$16T_{h}+9T_{em}\approx 0.816+25.632=26.448$ ms
Qureshi and Munir [13]	$2T_{aes}$	$2T_{em}$	$2T_{aes}+2T_{em}\approx 0.024+5.696+1=5.72$ ms
Wang et al. [14]	$6T_{h}$	$7T_{h}$	$13T_{h}\approx 0.663$ ms
Ours	$9T_{h}+1T_{em}$	$6T_{h}+3T_{em}$	$15T_{h}+4T_{em}\approx 0.765+11.392=12.157$ ms

**Table 5:** A Comparative Analysis: Communication Cost.

Scheme	Handshake	Total Costs
Kumari et al. [6]	3	1760 bits
Karuppiah et al. [10]	2	2848 bits
Huang et al. [16]	3	1600 bits
Jiang et al. [17]	2	1984 bits
Bhuarya et al. [11]	3	1760 bits
Qureshi and Munir [13]	7	2400 bits
Wang et al. [14]	5	3200 bits
Ours	3	1760 bits

**Table 6:** A Comparative Analysis: Storage Cost.

Scheme	Total Costs
Kumari et al. [6]	480 bits
Karuppiah et al. [10]	3712 bits
Huang et al. [16]	320 bits
Jiang et al. [17]	640 bits
Bhuarya et al. [11]	640 bits
Qureshi and Munir [13]	800 bits
Wang et al. [14]	960 bits
Ours	960 bits

**Table 7:** Execution cost (milliseconds).

Operation	Max. Time (ms)	Min. Time (ms)	Average Time (ms)
$T_{em}$	2.920	2.766	2.848
$T_{h}$	0.142	0.022	0.051
$T_{modexp}$	4.649	1.746	3.146
$T_{aes}$	0.021	0.011	0.012

$T_{em}$: elliptic curve scalar multiplication; $T_{h}$: hash function; $T_{modexp}$: modular exponentiation; $T_{aes}$: AES-256.

## Data Availability

Not applicable.
